# Supplementary material for: Geographical variation and determinants of women unemployment status in Ethiopia; A multilevel and spatial analysis from 2016 Ethiopia Demographic and Health Survey data
Source: PLoS One. 2022 Jul 7;17(7):e0270989. doi: 10.1371/journal.pone.0270989 (PMC9262193; doi:10.1371/journal.pone.0270989)
Supplement: S1 Table — (DOCX) [file pone.0270989.s001.docx]

**Supporting Information**

**Table S1:** Significant SaTScan spatial scan clusters for unemployment women across region in Ethiopia, 2016 EDHS

| **Type of cluster** | **Significant Enumeration Areas (clusters) detected** | **Total # of population** | **Total # of cases** | **RR** | **LL** | **Coordinates**  **/Radius** | **P-value** |
| --- | --- | --- | --- | --- | --- | --- | --- |
| Most likely cluster | 199,628,152,327,66, 163, 132, 312, 542, 512, 627, 80, 322,640,300, 401, 158,425,591,136, 638,456, 545, 392, 478, 38, 143, 97, 351,200,169, 449, 73, 538, 442, 455, 424,575, 292, 551, 188,579, 160, 167, 128,431,403, 340, 24,120,79,249,258,354,382,496,488,410,156,176,516,429, 237, 550, 612, 94,430,616,279,605,384,332,636,344,355,296, 189,220, 206, 421, 241,511, 617,460, 181, 611, 584, 597, 571, 18, 400,389, 130, 583, 81,590,98,504,345,604,191,481, 623, 361, 10, 544, 255, 99, 172, 84, 298, 129,226, 482, 528, 254, 45, 461, 348, 267, 259, 375,109, 599, 196, 3,341,229,253,78,602,474,368,127,268,235,585,598, 362,531, 89, 404, 541,350, 479, 510, 55, 263, 415,515,52,117,192,548,386,615,498,218,413,134,310,547,103, 572,570, 276, 620, 637,533, 246, 494, 205,423, 334, 559, 283,427, 499, 178, 36, 295, 102 | 3577 | 2388 | 1.43 | 200.78 | (12.376936 N,38.357984 E) / 318.07 km | <0.0001 |
| Secondary cluster^b^ | 446,270, 219, 284, 593, 417, 265, 13, 448, 106, 326 | 188 | 151 | 1.53 | 31.11 | (7.640999 N,34.491732 E) / 64.20 km | <0.0001 |
| Secondary cluster^b^ | 333,491, 412, 506, 93, 476, 372, 564, 39, 51, 441 | 207 | 151 | 1.39 | 17.67 | (9.299405 N,40.968436 E) / 86.81 km | <0.0001 |
| Secondary cluster^b^ | 168,552,465,459,371, 243, 526, 197, 299, 46 | 193 | 135 | 1.33 | 11.78 | (7.223847 N,35.325109 E) / 20.48 km | 0.0035 |
| Secondary cluster^b^ | 436, 212, 501, 622, 68, 454 | 116 | 86 | 1.41 | 11.09 | (9.301797 N,42.243309 E) / 9.43 km | 0.0066 |
| Secondary cluster^b^ | 82,7,601,377, 394, 422, 398, 208, 21, 316 | 197 | 134 | 1.29 | 9.379291 | (3.621391 N,39.291912 E) / 278.20 km | 0.049 |
