# Supplementary material for: Geographical variation and determinants of women unemployment status in Ethiopia; A multilevel and spatial analysis from 2016 Ethiopia Demographic and Health Survey data
Source: PLoS One. 2022 Jul 7;17(7):e0270989. doi: 10.1371/journal.pone.0270989 (PMC9262193; doi:10.1371/journal.pone.0270989)
Supplement: Attachment — Submitted filename: 2 nd revisoin plos.docx [file pone.0270989.s002.docx]

First of all thanks for your worth and essential over all comments. On the very beginning I am glad for your nice reviewing and assessing this article, entitled, Geographical variation and determinants of women unemployment status in Ethiopia; A multilevel and spatial analysis from 2016 Ethiopia Demographic and Health Survey data.

**Comment 1:** Your ethics statement should only appear in the Methods section of your manuscript. If your ethics statement is written in any section besides the Methods, please move it to the Methods section and delete it from any other section. Please ensure that your ethics statement is included in your manuscript, as the ethics statement entered into the online submission form will not be published alongside your manuscript.

**Response**: Thank you for your suggestion. we have incorporated the points that the ethical statement is only appear in the method section of the manuscript.

**Comment 2**. We note that Figures 4.1,4.2 and 4.3 in your submission contain map images which may be copyrighted. All PLOS content is published under the Creative Commons Attribution License (CC BY 4.0), which means that the manuscript, images, and Supporting Information files will be freely available online, and any third party is permitted to access, download, copy, distribute, and use these materials in any way, even commercially, with proper attribution. For these reasons, we cannot publish previously copyrighted maps or satellite images created using proprietary data, such as Google software (Google Maps, Street View, and Earth). For more information, see our copyright guidelines: [http://journals.plos.org/plosone/s/licenses-and-copyright](http://journals.plos.org/plosone/s/licenses-and-copyright).

We require you to either (1) present written permission from the copyright holder to publish these figures specifically under the CC BY 4.0 license, or (2) remove the figures from your submission:

A. You may seek permission from the original copyright holder of Figures 4.1,4.2 and 4.3 to publish the content specifically under the CC BY 4.0 license. 

We recommend that you contact the original copyright holder with the Content Permission Form ([http://journals.plos.org/plosone/s/file?id=7c09/content-permission-form.pdf](http://journals.plos.org/plosone/s/file?id=7c09/content-permission-form.pdf)) and the following text:
“I request permission for the open-access journal PLOS ONE to publish XXX under the Creative Commons Attribution License (CCAL) CC BY 4.0 ([http://creativecommons.org/licenses/by/4.0/](http://creativecommons.org/licenses/by/4.0/)). Please be aware that this license allows unrestricted use and distribution, even commercially, by third parties. Please reply and provide explicit written permission to publish XXX under a CC BY license and complete the attached form.”

Please upload the completed Content Permission Form or other proof of granted permissions as an "Other" file with your submission.

In the figure caption of the copyrighted figure, please include the following text: “Reprinted from [ref] under a CC BY license, with permission from [name of publisher], original copyright [original copyright year].”

B. If you are unable to obtain permission from the original copyright holder to publish these figures under the CC BY 4.0 license or if the copyright holder’s requirements are incompatible with the CC BY 4.0 license, please either i) remove the figure or ii) supply a replacement figure that complies with the CC BY 4.0 license. Please check copyright information on all replacement figures and update the figure caption with source information. If applicable, please specify in the figure caption text when a figure is similar but not identical to the original image and is therefore for illustrative purposes only.
The following resources for replacing copyrighted map figures may be helpful:

USGS National Map Viewer (public domain): [http://viewer.nationalmap.gov/viewer/](http://viewer.nationalmap.gov/viewer/)
The Gateway to Astronaut Photography of Earth (public domain): [http://eol.jsc.nasa.gov/sseop/clickmap/](http://eol.jsc.nasa.gov/sseop/clickmap/)
Maps at the CIA (public domain): [https://www.cia.gov/library/publications/the-world-factbook/index.html](https://www.cia.gov/library/publications/the-world-factbook/index.html) and [https://www.cia.gov/library/publications/cia-maps-publications/index.html](https://www.cia.gov/library/publications/cia-maps-publications/index.html)
NASA Earth Observatory (public domain): [http://earthobservatory.nasa.gov/](http://earthobservatory.nasa.gov/)
Landsat: [http://landsat.visibleearth.nasa.gov/](http://landsat.visibleearth.nasa.gov/)
USGS EROS (Earth Resources Observatory and Science (EROS) Center) (public domain): [http://eros.usgs.gov/#](http://eros.usgs.gov/)
Natural Earth (public domain): [http://www.naturalearthdata.com/](http://www.naturalearthdata.com/)

**Response**: Thank you for your suggestion. We incorporate your worth comments and the map images are not copyrighted,which are my work.

**Comment 3.** Thank you for submitting the above manuscript to PLOS ONE. During our internal evaluation of the manuscript, we found significant text overlap between your submission and the following previously published works.

- [https://bmcpublichealth.biomedcentral.com/articles/10.1186/s12889-019-7529-z](https://bmcpublichealth.biomedcentral.com/articles/10.1186/s12889-019-7529-z)
- [https://bmjopen.bmj.com/content/9/4/e027276](https://bmjopen.bmj.com/content/9/4/e027276)
- [https://pubmed.ncbi.nlm.nih.gov/24411023/](https://pubmed.ncbi.nlm.nih.gov/24411023/)

We would like to make you aware that copying extracts from previous publications, especially outside the methods section, word-for-word is unacceptable. In addition, the reproduction of text from published reports has implications for the copyright that may apply to the publications.

Please revise the manuscript to rephrase the duplicated text, cite your sources, and provide details as to how the current manuscript advances on previous work. Please note that further consideration is dependent on the submission of a manuscript that addresses these concerns about the overlap in text with published work.

We will carefully review your manuscript upon resubmission, so please ensure that your revision is thorough.

**Response:** Thank you for your suggestion. We incorporate your worth comments and we revise and paraphrase all the manuscript in detail.
