# Supplementary material for: Geographical variation and determinants of women unemployment status in Ethiopia; A multilevel and spatial analysis from 2016 Ethiopia Demographic and Health Survey data
Source: PLoS One. 2022 Jul 7;17(7):e0270989. doi: 10.1371/journal.pone.0270989 (PMC9262193; doi:10.1371/journal.pone.0270989)
Supplement: Attachment — Submitted filename: Response_2 for plos one.docx [file pone.0270989.s003.docx]

First of all thanks for your worth and essential over all comments. On the very beginning I am glad for your nice reviewing and assessing this article, entitled, Geographical variation and determinants of women unemployment status in Ethiopia; A multilevel and spatial analysis from 2016 Ethiopia Demographic and Health Survey data.

**Reviewer #1**:I am glad to review this exciting article . I directly recommend this study for publication . Fully satisfied

**Response**: We authors owe you a great debt of gratitude.

**Reviewer #2:** I appreciate that the authors have tried to clarify some of my previous comments. But my comments have not addressed no proper argument has been provided to justify their choices. I do not see any contribution in finding spatial correlation in unemployment: I doubt that in any country one can find that unemployment is randomly distributed across regions (which are endogenously formed in a way clearly linked to unemployment). The authors' response has ignored my point about the incorrect use of the word "outliers" as the authors do: this is not a standard way to name that situation, a better one exists and it is confusing. I do not see again that my comments on the second part of the paper (factors associated with women unemployment) have been addressed. As I said, this provides some mere correlations between factors and unemployment. This descriptive evidence can have some interest (this is an editorial choice), but it should be properly carried out AND it must be clear that NO CAUSAL interpretation should be concluded from it. On the one hand, if the authors carefully read my previous previous, I mention that STANDARD ERRORS should be account for clustering; I did not mention at all, as the authors focus on their reply, on using single-level or multilevel statistical models. If standard errors are not properly computed, results are not credible. On the other hand, the authors should be much clearer about the merely descriptive implications of their results. They cannot claim (as they implicitly and explicitly do) that a changing some factors (eg., improving entrepreneurship,etc.) could have an effect on unemployment: this a causal claim that cannot be derived from the analysis and this should be clear in a serious scientific paper.

**Response**: At the outset, we want to thank you for taking the time to review and evaluate this article again.

The research is routinely used to demonstrate the initial goal of investigating the spatial structure of unemployment rate across different clusters to provide implications for policymakers, investigating the hot spots of unemployment rate, and showing a visual picture of unemployment rate. Mapping was used as a preliminary step in conducting a visual inspection for the unemployment rate. **In addition, mapping is important in the monitoring of unemployed people. Maps can reveal spatial patterns that were previously unknown or unnoticed when examining a table of statistics, as well as high-risk communities or problem areas. The goal of spatial analysis is to find patterns in geographic data and try to explain them**. **Spatial autocorrelation is the term used for theinterdependence of the values of a variable over space.As a result, the most important contribution in determining spatial correlation/statistics in unemployment is not only interested in answering the "how much unemployment rate women in Ethiopia" question, but also the "how much is where" question.** As stated in the first law of geography, "everything is related to everything else, but closer things are more related than distant things," the application of statistical techniques to spatial data faces significant challenges. Two spatial autocorrelation statistics based on sharing boundary neighbors, known as global and local Moran's I, were used to investigate global clustering and local clusters, respectively. Based on visual inspection of the mapping, global clustering was discovered in unemployment rate, which was confirmed by the significant statistic discovered by global Moran's I(Moran’s *I* values I=0.33 (p-value=0.001).

Our interest is frequently not only in determining whether the data as a whole exhibits spatial auto-correlation, but also in identifying specific observations that exhibit spatial auto-correlation with their neighbors. As a result, the study relied on local measures known as local spatial auto-correlation indicators (LISA), which are a collection of local indicators used to determine the scope of clustering regions (in our case enumeration areas). Once a significance level has been determined (if the global spatial auto-correlation is significant), values can be plotted on a map to show the precise locations of hot spots and potential outliers (dissimilar values) or A positive local Moran value indicates local stability, such as a cluster with a high/low unemployment rate surrounded by another cluster with a high/low unemployment rate. A negative local Moran value indicates local instability(high/low outlier ), such as a cluster with low unemployment surrounded by a cluster with high unemployment, or vice versa. Figures 2 and 3 show the results of the LISA map.

And ,Thank you for your suggestion and comments. This is a very essential comment, we have incorporated the points. We repeatedly read the whole document and consider all the corrections.

After all, concluding that the spatial distribution of women's unemployment status at the national level is non-random (I=0.33; p-value=0.001) and associated with neighboring values. The result indicates that there is spatial autocorrelation /dependency or the unemployment rate is interdependent across clusters. As a result, we employ a multilevel logistic regression model that accounts for a lack of independence (spatial correlation) across levels of nested data (i.e., individuals (women) nested within clusters). The spatial correlation result was also supported by the intra-class correlation coefficient (ICC), which measures the proportion of variance in the outcome explained by the grouping structure while accounting for variance across clusters. This ICC represents the correlation of the women's unemployment rate within the same enumeration areas, i.e. the dependency of the unemployment rate among women within the clusters.So,from the research analysis, About 21.48% of the variation in women's unemployment rate occurred due to community-level(due to clusters) factors (ICC =21.48%).
