# Supplementary material for: Geographical variation and determinants of women unemployment status in Ethiopia; A multilevel and spatial analysis from 2016 Ethiopia Demographic and Health Survey data
Source: PLoS One. 2022 Jul 7;17(7):e0270989. doi: 10.1371/journal.pone.0270989 (PMC9262193; doi:10.1371/journal.pone.0270989)
Supplement: Attachment — Submitted filename: Response_3_ for plos one.docx [file pone.0270989.s004.docx]

**Response to Reviewers**

First of all thanks for your worth and essential over all comments. On the very beginning I am glad for your nice reviewing and assessing this article, entitled, Geographical variation and determinants of women unemployment status in Ethiopia; A multilevel and spatial analysis from 2016 Ethiopia Demographic and Health Survey data.

Reviewer #2: I do not see any real improvement with respect to the previous versions. None of my comments that the editor mentioned have been properly addressed.

I feel that a crucial point in any serious scientific paper as making clear that the results cannot be interpreted in a causal way CANNOT be addressed just by adding a "could be". Instead it should be explicitly mentioned the true purely descriptive scope of the results.

The authors do not only insist in using the term "outlier" in a unconventional sense, but they have now extended its use. Even more surprisingly they do not even attempt to provide a simple explanation about their choice, although I have mentioned this in my two previous reports. It is really a complete outlier the way in which the author insist in using the term "outlier" (positive correlation between two variables, say, high education and high income is never called a positive outlier). And the way in which the authors completely ignore this point (to accept it or to provide a convincing alternative explanation about their unusual way of using the term) is more outlier in peer review process.

I perfectly know that the interesting question is to show where the unemployment is located and how is related to nearby areas. I never raised this point that the authors insist in explaining while ignoring other more important and explicitly mentioned. However, the paper is more focused precisely in trivial and uninteresting things: to discussed the global Moran statistics that reveals the TRIVIAL point that unemployment is not uniform across regions (randomly distributed) as I already mentioned.

I perfectly know how to interpret the local spatial auto-correlation indicators. If the authors carefully read and understand this and my previous reports, they would see that the literature in this area NEVER calls OUTLIERS to what is simply positive or negative correlation amongst clusters (high/high, high/low, etc.). The term outliers is NOT used in this or any other literature to name dissimilar values (high/low); and, as I already mention, the standard output of statistical software already provides a proper term for this (and it is not outlier). Even more, if the authors were using outlier in the sense of dissimilar values (which is unconventional and not justified or explained by the authors), high-high and low-low cases are also identified as "outliers" but in this case the values are not dissimilar...

Finally the authors have completely ignored (again) my point about standard errors. Statistical modelling (i.e., using a multilevel model or not) is not the only relevant part of empirical results; and in any case it was never my point. In any empirical papers, researcher should be very careful about inference. And in this case it means to use proper standard errors. Otherwise, the results could be meaningless: some factors that the authors claim to be correlated to unemployment might actually be not significantly related to it. Once again the authors do not even mention why this crucial point is not even properly addressed..

**Response:** Thank you for your suggestion and comments. This is a very essential comment, we have incorporated the points. We repeatedly read the whole document and consider all the corrections.

At the outset, we want to thank you for taking the time to review and evaluate this article again. The manuscript was revised (all the spatial output was updated). Your comment is clear but please understated the aim of this study. This research is routinely used to assess the spatial distribution (variation) of unemployment rate across different clusters to provide implications for policymakers, investigating the hot spots of unemployment rate(the highest risk region/areas), and showing a visual picture of unemployment rate in the first section.

In this part we identify that:

Spatial pattern of women unemployment

In Ethiopia, the spatial distribution of women's unemploymentwas spatially clustered with Global Moran's I = 0.064298 (z-score = 16.375386, P-value <0.0001). This demonstrated that spatial hotspot and coldspot clustering was identified in Ethiopian regions. Given the z-score of 16.375386, there was less than a 1% chance that this high-clustered pattern was the result of random chance. The bright red and blue colors on the tails indicate a higher level of significance (Fig. 2).

Then we find the hot spot also:

Hot spot (Getis-Ord Gi*) analysis

Figure3 summarizes a hot and cold spot analysis of the risk locations for women's unemployment in Ethiopia. Beneshagul and Gambela, Oromia, WesternAmhara, and northeren SNNPR are the regions with the highest unemployment rates. Tigray, Afar, Dire Dawa, Hariri, northern Amhara, and Somalia on the other hand, have been designated as cold-spot (low risk) regions (Fig 3).

And we can identify the most likely cluster by Spatial scan statistics:

Spatial SaTScan analysis of unemployment women across region

Most likely (primary clusters) and secondary clusters of unemployment women were identified. In 2016 EDHS, spatial scan statistics identified a total of high and modest performing spatial clusters of unemployment women. A total of 164 significant clusters were identified. Of these, among the significant clusters, 120 were most likely (primary cluster) and the other 44 were secondary. The spatial window of the primary cluster was located at Tigray, Amhara, southern Gambela,eastern Oromia and Afar which was centered at (12.376936 N,38.357984 E) / 318.07 km, RR = 1.43, and log likelihood ratio (LLR) of 200.78 at p-value < 0.0001. It showed that house holds in the spatial window had 1.43 times higher unemployment women ratethan those outside the window (Table 2 and Fig. 4).

Here we are addressing the spatial distribution and the risk area/cluster of unemployment, which means we speak about the first aim of this study.

In the next section of this study: We need to identify the determinant factors of women unemployment rate in Ethiopia

After all, concluding that the spatial distribution of women's unemployment status at the national level is non-random and associated with neighboring values. The result indicates that there is spatial autocorrelation /dependency or the unemployment rate is interdependent across clusters. As a result, we employ a multilevel logistic regression model that accounts for a lack of independence (spatial correlation) across levels of nested data (i.e., individuals (women) nested within clusters). The spatial correlation result was also supported by the intra-class correlation coefficient (ICC), which measures the proportion of variance (indirectly we know the standard deviation) in the outcome explained by the grouping structure while accounting for variance across clusters. This ICC represents the correlation of the women's unemployment rate within the same enumeration areas, i.e. the dependency of the unemployment rate among women within the clusters. So, from the research analysis, About 21.48% of the variation in women's unemployment rate occurred due to community-level (due to clusters) factors (ICC =21.48%).and MOR is showing that there was variation between communities clustered. So, here we know that the within and between cluster variation.

The result and interpretation was clearly mentioned in the manuscript.

Next, we examined the factors that influenced Ethiopia's unemployment rate using MLM, since this model incorporates the cluster correlation between unemployment and various factors.
