# Supplementary material for: Geographical variation and determinants of women unemployment status in Ethiopia; A multilevel and spatial analysis from 2016 Ethiopia Demographic and Health Survey data
Source: PLoS One. 2022 Jul 7;17(7):e0270989. doi: 10.1371/journal.pone.0270989 (PMC9262193; doi:10.1371/journal.pone.0270989)
Supplement: Attachment — Submitted filename: point by point response to women unemployment R4.docx [file pone.0270989.s005.docx]

**Thank you for your comments**

Journal Requirements:

Please review your reference list to ensure that it is complete and correct. If you have cited

papers that have been retracted, please include the rationale for doing so in the manuscript text,

or remove these references and replace them with relevant current references. Any changes to

the reference list should be mentioned in the rebuttal letter that accompanies your revised

manuscript. If you need to cite a retracted article, indicate the article’s retracted status in the

References list and also include a citation and full reference for the retraction notice.

**Response:** Please accept our sincere thanks for your comment. We carefully review our reference list and ensure that it is accurate. Additional references are also added to the list.

31. Broussard, N. and T.G. Tekleselassie, *Youth unemployment: Ethiopia country study.* International Growth Centre. Working Paper, 2012. **12**(0592): p. 1-37.

32.	Nganwa, P., D. Assefa, and P. Mbaka, *The nature and determinants of urban youth unemployment in Ethiopia.* Nature, 2015. **5**(3): p. 197-203.

33.	Batu, M.M., *Determinants of youth unemployment in urban areas of Ethiopia.* International Journal of Scientific and Research Publications, 2016. **6**(5): p. 343-350.

Reviewer #2:

 I do not see how my points were at odds with the aim of the study. Identifying hot spots areas is fine, but one cannot use concepts (i.e., outlier) in a completely different way as the literature does or one must provide good arguments for such unconventional choice. The authors have gone much beyond this modest point and now provide a completely new set of results. While I did not ask to follow this approach, I acknowledge that it has improved a lot the text and I prefer these results to the previous ones even if they had included my proposed corrections. The current results are in the line with the previous ones, although they are more complete and provide better insights.

In the previous version, the authors defined/used "outlier" in an incorrect way according to the literature. Now the term "outlier" is only used a couple of times in the Discussion section, but never properly defined. An "outlier" does not mean any value just above or below the mean or a reference value, but a really extreme value. It is never clear in the main text how an outlier is defined; for instance, how many times apart from mean/reference value and WHY, as it is routinely done in any rigorous statistical publication dealing with outliers.

**Response:** Please accept our sincere thanks for your comment. All your comments have been incorporated into the current version of the manuscript.
